# Morphological, molecular and ecological characterization of a native isolate of *Steinernema feltiae* (Rhabditida: Steinernematidae) from southern Chile

**DOI:** 10.1186/s13071-020-04548-7

**Published:** 2021-01-13

**Authors:** Patricia Flores, Andrea Alvarado, Gabriela Lankin, Paola Lax, Simona Prodan, Erwin Aballay

**Affiliations:** 1 Departamento de Sanidad Vegetal, Facultad de Ciencias Agronómicas, Universidad de Chile, P.O. Box 1004, Santiago, Chile; 2 Instituto de Diversidad y Ecología Animal (CONICET-UNC) y Centro de Zoología Aplicada, Facultad de Ciencias Exactas, Físicas y Naturales, Universidad Nacional de Córdoba, X5000AVP Córdoba, Argentina

**Keywords:** Biocontrol, Symbiotic bacteria, Juveniles, Adults, Taxonomy, *Xenorhabdus*, Temperature, Soil water content

## Abstract

**Background:**

*Steinernema feltiae* is an entomopathogenic nematode used in biological control programs with a global distribution. Populations of this species show phenotypic plasticity derived from local adaptation and vary in different traits, such as location and host penetration. The aim of this work was to describe a Chilean isolate of this nematode species, using integrative approaches.

**Methods:**

Nematode morphological and morphometric studies were conducted along with molecular analysis of nuclear genes. The symbiotic bacterium was also identified by sequencing the 16S rRNA gene. Some ecological characteristics were described, including the temperature requirements for the nematode life cycle and the effect of soil water content for optimal reproduction.

**Results:**

Morphometric characterization revealed a large intra-specific variability. The isolate identity was also corroborated with the analysis of nuclear genes. Based on the 16S gene, its symbiont bacteria, *Xenorhabdus bovienii,* was identified. The lowest, optimal and highest temperatures found to limit the infestation and reproduction on *Galleria mellonella* were 10, 20 and 30 °C, respectively; the emergence from the host larvae occurred approximately 10 days after inoculation. Differences were observed in offspring, and 120 infective juveniles (IJ)/larva was the most prolific dose at 20 °C. The soil water content did not affect the number of IJ invaders, penetration efficacy and IJ emergence time or offspring per larva, but it caused a delay in achieving full mortality at the permanent wilting point with respect to saturation and field capacity.

**Conclusions:**

For the first time, a Chilean isolate of *S. feltiae* is described in detail considering morphological, molecular and ecological aspects. The isolate was shown to be efficient in soil containing water, with optimal temperatures ranging from 15 to 25 °C for host infestation and production of an abundant offspring; these characteristics would allow its potential use as control agents in a wide geographical area of the country.
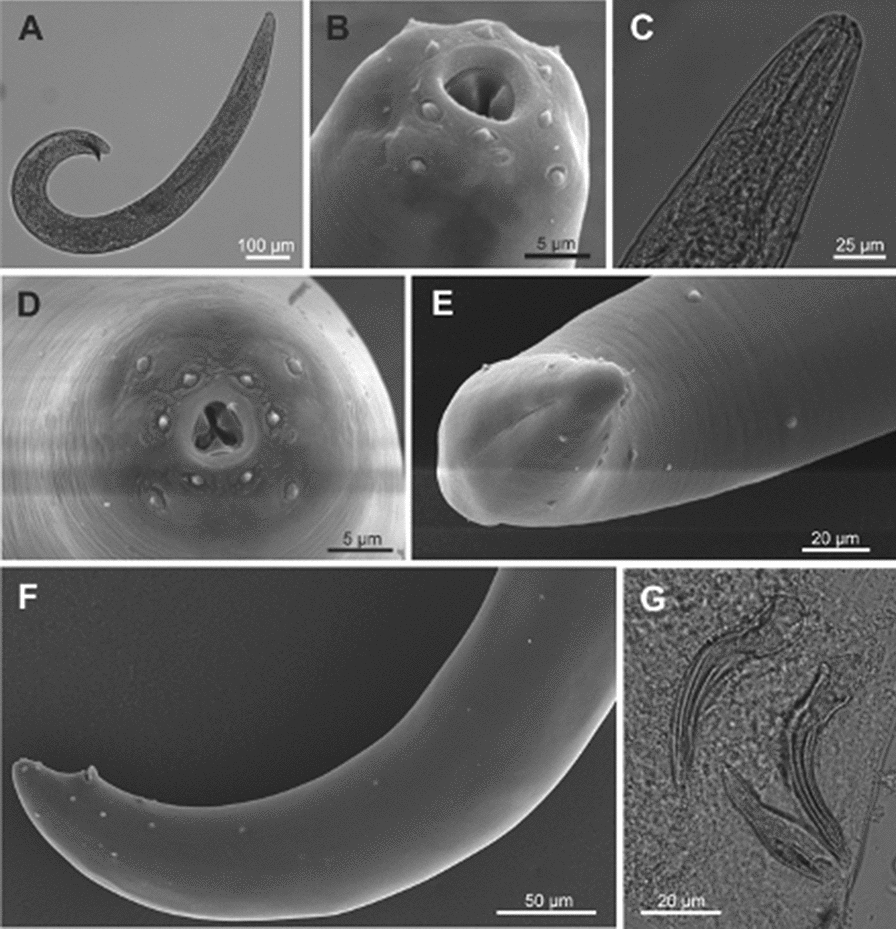

## Background

Entomopathogenic nematodes (EPNs) are lethal insect parasites that belong to the families Steinernematidae and Heterorhabditidae; its infective juveniles (IJs) carry symbiotic bacteria of the genera *Xenorhabdus* and *Photorhabdus*, respectively, within their intestines. The search for new alternatives for pest management has promoted new surveys and research in the area of biological control. The potential of EPNs for the control of insects continues to be one of the most studied alternatives, as reflected, for example, in the rate of new descriptions [1]. This surge in interest stems from the need to evaluate more intensively their potential to replace some synthetic insecticides for the control of soil-borne pests. These nematodes appear to be good insect control agents considering their easy mass production, wide host range [2] and relative safety with respect to nontarget organisms and the environment [3]. On the other hand, antimicrobial and insecticidal compounds, particularly from symbiotic bacteria, have also received special attention [4, 5].

A better understanding of the biology and ecology of EPN species can improve the effectiveness of biological control. It has been shown that populations of the same species from different locations may have phenotypic plasticity derived from local adaptation and may vary in different traits, such as location and host penetration capacity [6]. These behaviors are influenced by abiotic and biotic factors as well as by intrinsic nematode characteristics [7]. Knowledge of key intrinsic and extrinsic factors affecting the infection process can help optimize the management of EPNs for biological control [8].

The search for and study of EPNs in Chile is a relatively new discipline. The country has a high diversity of ecosystems, suggesting a rich fauna adapted to different environmental conditions [9]. Previous surveys detected three new species, *Heterorhabditis atacamensis* [10], *Steinernema unicornum* [11] and *Steinernema australe* [12], and several *Steinernema feltiae* isolates from different habitats [9]. The *S. feltiae* specimens were identified by molecular assessment of the internal transcribed spacer (ITS) rDNA region. So far, there is no complete description of any isolate of this species from Chile, including ecological aspects. For this reason, the aim of this study was to describe morphological and molecular characteristics of a new isolate, identify its symbiotic bacteria and assess some environmental requirements for the nematode life cycle under different soil and climate conditions.

## Methods

### Nematode isolate and symbiotic bacterium culture

The *S. feltiae* isolate Lican Ray (LR) was recovered from soil obtained from an oak forest near the city Lican Ray (39°28′12″ S, 72° 7′ 12″ W) by baiting the soil with greater wax moth larvae, *Galleria mellonella* [13]. Glass flasks of 500 cm^3^ were closed and kept at 20 °C for 96 h; the dead insects were moved to modified White traps [14], and emerging IJs were collected to infect new insect larvae to increase the population. Emergent IJs were then stored in tap water at 10 °C.

To retrieve symbiotic bacteria, a pool of IJs was surface-sterilized in 2% NaClO for 3 min, washed thoroughly with sterile water and crushed to release the bacteria. One aliquot of the homogenate was streaked onto plates with nutrient agar supplemented with 0.004% (w/v) triphenyltetrazolium chloride and 0.025% (w/v) bromothymol blue, pH 7 (NBTA plates) [15, 16]. On this solid culture medium, *Xenorhabdus* colonies exhibit a typical green-blue color, which allows them to be distinguished from potential contaminants [17]. After 48 h of incubation at 28 °C, colonies corresponding to the symbiotic phenotype were isolated and preserved at − 80 °C in nutrient broth supplemented with 20% glycerol.

### Morphological and morphometric studies

First- and second-generation adults and IJs were collected at random from infected insect larvae [14]. Males and females were collected on the 4th and 8th days after inoculation of *G. mellonella* for the first and second generations, respectively, while IJs were collected within 2 days after emergence. For descriptive purposes, 25 specimens for each stage were fixed in TAF and processed to glycerin by Seinhorst’s rapid method [18]. Morphological and morphometric parameters suggested by Hominick et al. [19] were analyzed using an Axiocam MRC in a Zeiss Axioimager A.1 light microscope.

For scanning electron microscopy, adults were obtained from dead *G. mellonella* larvae and IJs were recovered from White traps; all specimens were washed thrice in buffer M9. All nematodes were relaxed in 60 °C water, fixed in 8% glutaraldehyde and mounted according to the methodology of Koppenhöfer and Stock [20]. Scanning was performed using a Philips XL microscope with an SES DS-130 at 20 kV accelerating voltage.

### Molecular characterization

#### Nematode isolate

DNA was obtained from single females using the extraction method of Williams et al. [21]. Nematodes were collected in PCR tubes with WLB buffer containing 10 mg/ml proteinase K and frozen for at least 10 min at − 80 °C. The tubes were quickly placed in a water bath at 65 °C and then incubated at the same temperature for 90 min to allow digestion by proteinase K. Finally, proteinase K was inactivated by heating to 95 °C for 15 min, and the tubes were centrifuged to separate the supernatant.

PCR was performed to amplify the large ribosomal subunit (LSU) 28S rDNA using forward primer no. 391 (5′-AGCGGAGGAAAAGAAACTAA-3′) [22] and D3B (5′-TCGGAAGGAACCAGCTACTA-3′) reverse primer [23]. One fragment of rDNA that includes the internal transcribed spacer ITS-1, the 5.8S subunit and ITS-2 was PCR amplified using the primer pair 93 (5′-TTGAACCGGGTAAAAGTCG-3′) and 94 (5′-TTAGTTTCTTTTCCTCCGCT-3′) [24]. In both PCRs, a volume of 2 µl DNA was used as template in a 50 µl reaction mix that contained 0.5 µM of each primer, 200 µM dNTP and 1 unit of Taq DNA Polymerase Recombinant (Invitrogen) along with 1.5 mM MgCl_2_ final concentration. Amplifications were performed in a BIOER-LifePro Thermal Cycler. To amplify the LSU fragment, the PCR mix was denatured at 94 °C for 3 min, followed by 33 cycles of 94 °C for 30 s, 52 °C for 30 s and 72 °C for 1 min, and a final extension of 7 min at 72 °C. A similar PCR program was used for the ITS region, adjusting the annealing temperature to 60 °C. The amplified fragments were separated by electrophoresis on 1% agarose (w/v) gels using 1X TBE buffer at 100 V for 1 h and then purified using an E.Z.N.A. Gel Extraction Kit (OMEGA Bio-tek). PCR products were sequenced (Macrogen, USA) using internal primers. Forward 502 (5′-CAAGTACCGTGAGGGAAAGTTGC-3′) and reverse 503 (5´-CCTTGGTCCGTGTTTCAAGACG-3′) primers were used for 28S; forward 533 (5′-CAAGTCTTATCGGTGGATCAC-3′) and reverse 534 (5′-GCAATTCACGCCAAATAACGG-3′) were used for ITS fragment [25].

#### Symbiotic bacteria

DNA was extracted from 1.5 ml of overnight Miller’s LB Broth (10 g/l Tryptone, 10 g/l NaCl, 5 g/l yeast extract) culture, using a GenElute Bacterial Genomic DNA kit (Sigma, Sigma-Aldrich). Universal primers that amplify nearly the full-length 16S rRNA from many bacterial genera were used: 27f (5′-AGAGTTTGATCATGGCTCAG-3′) and 1492r (5′-TACGGTTACCTTGTTACGACTT-3′) [26]. The reaction was carried out in a final volume of 30 µl containing 1 µl DNA, 1 µM of each primer, 200 µM dNTP and 1 unit Taq DNA Polymerase Recombinant (Invitrogen). PCR parameters consisted of an initial denaturation at 94 °C for 3 min, 35 cycles of 94 °C for 50 s, annealing at 58 °C for 50 s and 72 °C extension for 50 s followed by a final extension at 72 °C for 7 min. PCR products were visualized, purified and sequenced as previously mentioned.

#### Phylogenetic analysis

The DNA sequences of the isolate LR were compared with those present in GenBank using the basic local alignment search tool (BLAST) of the National Center for Biotechnology Information (NCBI). The 28S and ITS sequences and corresponding reference nucleotide sequences of *Steinernema* spp., including the *feltiae* group, available in GenBank were aligned with the default parameters of Clustal W [27]. The alignments were manually edited using BioEdit [28]. The 16S sequence of the symbiotic bacteria was aligned to corresponding sequences of *Xenorhabdus* spp.

Phylogenetic analyses were performed with maximum likelihood (ML) based on the Tamura-Nei model [29] using the program Molecular Evolutionary Genetics Analysis Version 6.0 (MEGA 6) [30]. The estimation of the support for each node was assessed by bootstrap analysis with 1000 replicates. The dataset was also analyzed using Bayesian inference with MrBayes 3.1.2 [31]. The best fitted model of DNA evolution was obtained using jModelTest 0.1.1 [32] with the Akaike information criterion. The GTR + G model (ITS and 28S) and GTR+G+I models (16S) were selected. Two independent runs were performed simultaneously on the data, each one using one cold and three heated chains. After 5 million generations, the average standard deviations of split frequencies between the two independent runs at completion were 0.005 (ITS and 16S) and 0.006 (28S). After discarding 25% of burn-in samples and evaluating convergence, the remaining samples were retained for further analyses. The topologies were used to generate a 50% majority rule consensus tree. Posterior probabilities are given on appropriate nodes. Trees were visualized using TreeView [33]. The newly obtained sequences were submitted to the NCBI GenBank database under the accession numbers indicated in bold on the phylogenetic trees.

### Ecological characteristics

Different experiments were performed to determine the optimal conditions of the nematode life cycle.

#### Effect of temperature

The optimal temperature for insect mortality, IJ penetration rate, days to emerge from the insect cadaver and offspring production were determined. One hundred IJs were applied in 0.2 ml of water per *G. mellonella* larva in petri dishes (3.5 cm diameter) with a filter paper on the bottom. Plates were covered with a plastic bag to maintain humidity and stored in an incubator at different temperatures (5, 10, 15, 20, 25 and 30 °C). Each treatment had five replicates distributed in a random design; each replicate consisted of a group of four plates and four larvae per plate. Insects were checked during 5 days to determine mortality; the larva was considered dead when it did not respond to being touched with a needle. One day after death, the cadaver was dissected, and the number of IJs inside the body was counted to estimate the penetration rate. Offspring were determined by counting the total number of emerged IJs from dead larvae in a modified White trap. Nematodes were recovered and stored at 10 °C in Falcon tubes every day, until no new IJs were detected.

#### Lethal concentration

A similar assay allowed evaluating the same parameters and the IJ penetration efficacy at different inoculum densities. Each petri dish, containing one *G. mellonella* larva, was inoculated by applying different doses per larva (0, 10, 20, 40, 80 and 240 IJs/0.2 ml water). During 4 days, plates were incubated at 20 °C, with the optimal temperature determined on the previous assay. Penetration efficacy (%) was calculated according to the formula of Kaya and Stock [14], which relates the number of nematodes recovered from dissection with those inoculated. Recovered nematodes were maintained as indicated above. Mortality percentages at 48 h were used to estimate the lethal concentration (LC).

*Effect of soil water content:* Substrates containing three water levels, permanent wilting point, field capacity and saturation, were used to determine IJs infestation capacity. The substrate consisted of a steamed mixture of uniform proportions of sand, agricultural soil and organic matter, with a content of clay, silt and sand of 13.2, 22.9 and 63.9%, respectively. The texture was sandy loam. Petri dishes (3.5 cm diameter, with a filter paper on the bottom) containing 4.5 g of the substrate were inoculated with 120 IJs/0.2 ml water. This nematode density was selected according to assay 2.4.2; after 30 min, one *G. mellonella* larva was placed in each petri dish, stored under the conditions previously indicated and incubated at 20 °C. The experimental design and the variables evaluated were the same as those mentioned above.

### Data analysis

The three experiments were repeated twice under the same conditions. For all the assays, mortality was corrected according to Abbot’s formula [34]. The percentage data were arcsine transformed (angular transformation), and normality and variance homogeneity were verified prior to performing an ANOVA using the program Minitab V 15. The other variables were analyzed with no transformations. A Tukey test was performed in case the ANOVA showed significant differences (*P* < 0.05). To determine LC_50_ and LC_90_, mortality at 48 h was considered; data were analyzed using the Probit Program V 1.5.

## Results

### Morphological and morphometric studies (Table 1, Figs. 1, 2, 3)

#### Male, first generation

**Fig. 1:**
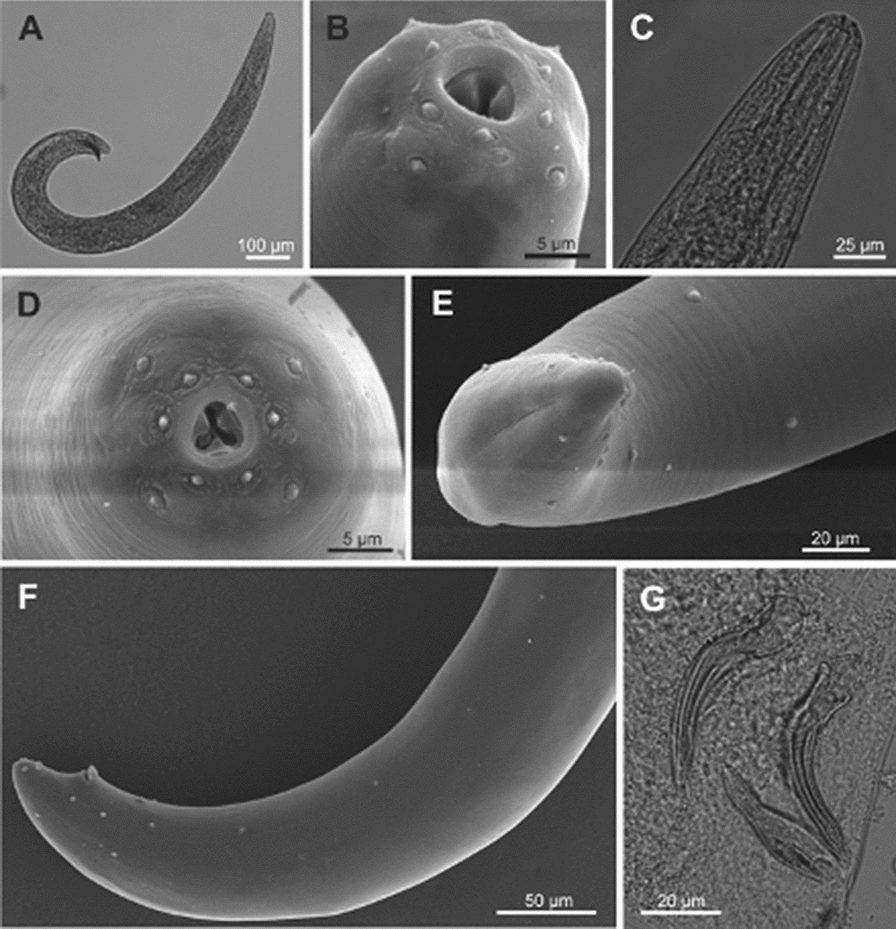
*Steinernema feltiae* isolate from Lican Ray, Chile. Male, first generation. **a** Entire body; **b**, **d** cephalic region, showing lips, labial papillae and cephalic papillae and amphids; **c** anterior region, showing excretory pore location; **e** tail with mucron; **f** posterior region, showing genital papillae; **f** spicules and gubernaculum

Body C- or J-shaped posteriorly when heat-killed (Fig. 1a). Cuticle with fine, annular striation under SEM but smooth under light microscope; lateral fields and phasmids inconspicuous. Anterior end slightly rounded, continuous with the body. Six prominent lips, each lip bearing a labial papilla. Four cephalic papillae, also notorious (Fig. 3b, d). Small amphidial opening, behind to lateral lip papillae. Deirids conspicuous, located in the first third, after than excretory pore. Stoma short and wide, inconspicuous sclerotized walls. Esophagous muscular with cylindrical procorpus, metacorpus slightly swollen, isthmus fairly notorious, ending in a pyriform basal bulb. Nerve ring surrounding the isthmus or the anterior end of basal bulb. Excretory pore anterior to the nerve ring, around first third of the isthmus (Fig. 1c). Simple testis, reflexed. Vas deferens with inconspicuous walls. Spicules paired, symmetrical, curved, ocher brown color (Fig. 1g); head (manubrium) oblong, shaft (calomus) notorious, velum present. Gubernaculum curved, approximately 2/3 of spicule’s length; boat-shaped in lateral view, anterior end curved ventrally (Fig. 1g); in ventral view, corpus with two projections. Tail conoid, tail terminus with a mucron (Fig. 1e). One single, midventral, precloacal papilla, and 11 pairs of papillae. Six pairs are precloacal, subventral, one pair lateral precloacal, one pair adanal, two pairs subterminal subventral and one pair post cloacal, lateral (Fig. 1f).

**Table 1:** Morphometrics of *Steinernema feltiae* isolate Lican Ray, Chile

Character	Male generation		Female generation		Third juvenile stage
	1°	2°	1°	2°	
*n*	25	25	25	25	25
L	1428 ± 86 (1309–1578)	866 ± 75 (731–1004)	5318 ± 907 (3856–7327)	3269 ± 286 (2890–3930)	807 ± 21 (779–841)
MBW	95 ± 7 (85–116)	53 ± 5 (44–64)	189 ± 27 (156–243)	154 ± 11 (131–173)	31 ± 4 (27–41)
ES	143 ± 10 (122–177)	115 ± 9 (99–138)	167 ± 17 (141–200)	166 ± 15 (136–200)	119 ± 7 (104–130)
EP	101 ± 9 (80–118)	72 ± 7 (61–86)	90 ± 17 (46–118)	85 ± 14 (67–117)	55 ± 3 (50–60)
NR	113 ± 11 (90–144)	85 ± 7 (71–101)	117 ± 16 (93–149)	114 ± 13 (86–140)	87 ± 5 (80–98)
TL	39 ± 3 (35–47)	35 ± 3 (31–40)	55 ± 6 (42–64)	48 ± 7 (37–62)	74 ± 3 (68–78)
ML	5 ± 1 (4–8)	11 ± 2 (8–14)			
ABD	40 ± 4 (35–48)	32 ± 3 (25–39)	65 ± 15 (42–91)	47 ± 6 (37–60)	16 ± 1 (14–20)
SpL	68 ± 5 (58–78)	57 ± 6 (42–65)			
GuL	46 ± 3 (40–55)	36 ± 4 (29–45)			
SW	1.7 ± 0.2 (1.3–2.1)	1.8 ± 0.3 (1.3–2.4)			
GS	0.7 ± 0.05 (0.6–0.8)	0.6 ± 0.1 (0.5–0.7)			
V			50 ± 2 (46–55)	53 ± 3 (49–58)	
a					26 ± 3 (19–31)
b					7 ± 0.4
c					11 ± 0.4 (10–12)
D%	71 ± 7 (56–84)	62 ± 6 (52–79)	54 ± 10 (33–67)	51 ± 7 (39–62)	46 ± 4 (40–55)
E%	261 ± 32 (202–326)	207 ± 27 (162–252)	165 ± 36 (76–232)	180 ± 44 (118–263)	75 ± 5 (67–83)
H					26 ± 4 (17–33)
H%					36 ± 6 (23–45)

Measurements are in µm, except the indexes, in form: mean ± standard deviation (range)

L: total body length; MBW: maximum body width; ES: esophagus length; EP: anterior end to excretory pore; NR: anterior end to nerve ring; TL: tail length; ML: mucron length; ABD: anal body diameter; SpL: spicule length; GuL: gubernaculum length; H: hyaline portion; V: position of vulva (%); D%: (EP/ES) × 100; E% = (EP/TL) × 100; SW = SpL/ABD; GS = GuL/SpL; H% = (H/TL) × 100; a = L/MBW; b = L/ES; c = L/TL

**Fig. 2:**
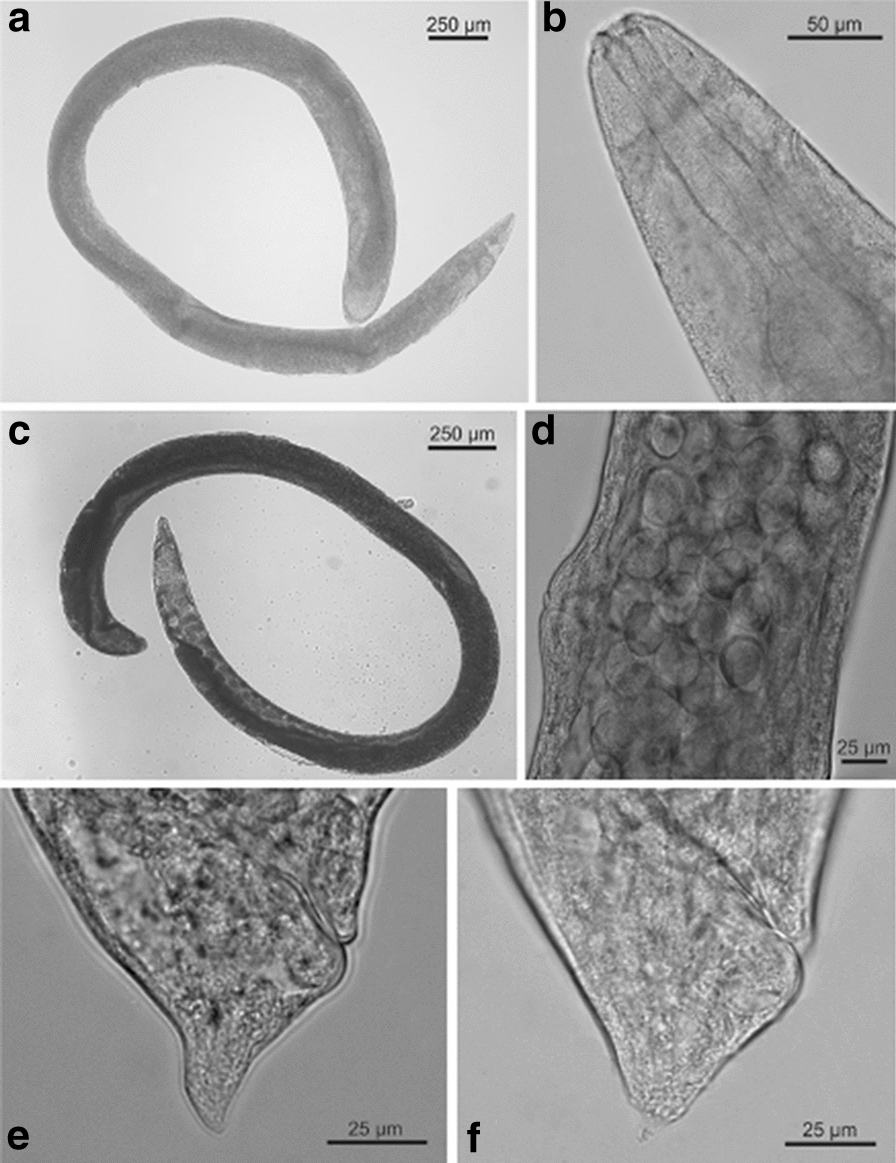
*Steinernema feltiae* isolate from Lican Ray, Chile. Female. First generation. **a** Entire body; **b** anterior region, showing esophagus and excretory pore. Second generation. **c** Entire body. First generation. **d** Vulvar region; **e** tail. Second generation. **f** Tail

**Fig. 3:**
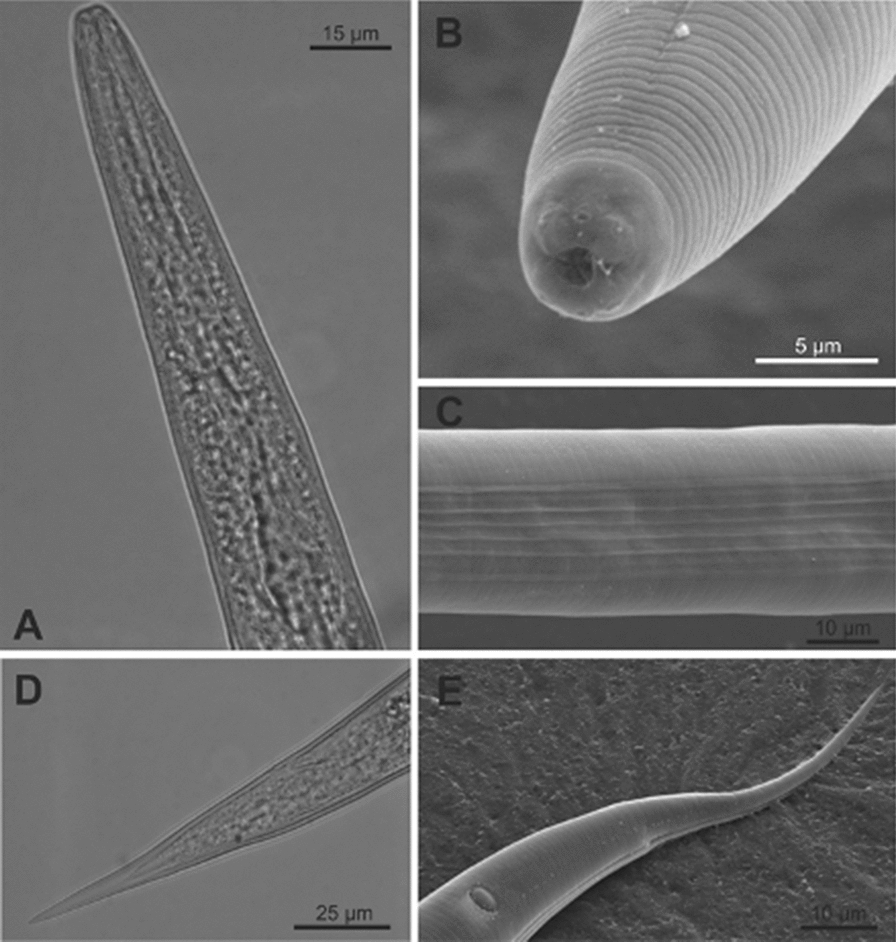
*Steinernema feltiae* isolate from Lican Ray, Chile. Third juvenile stage. **a** Anterior region, showing esophagus; **b** anterior region, showing cephalic papilla and amphid; **c** lateral field at midbody; **d** hyaline portion; **e** Tail

#### Male, second generation

Similar to the first-generation male, but more slender and smaller in body length and other morphometric characters. Deirids not observed. Mucron on tail terminus present and longer than that in the first generation.

#### Females, first generation

Body robust, habitus C-shaped (Fig. 2a). Cuticle, lips, stoma and esophageal region as in males. Excretory pore at mid of metacorpus (Fig. 2b). Reproductive system didelphic-amphidelphic, ovary reflexed dorsally. Vulva a transverse slit at midbody region, protuberant, with a double epiptygma (Fig. 2d). Vagina short, leading into paired uteri. Tail conoid with ventral postanal swelling (Fig. 2e). Mucron absent.

#### Females, second generation

Similar to first-generation females (Fig. 2c), but smaller in size. Vulva located slightly back compared to the first-generation females, symmetric and protuberant lips with a double epiptygma. Relation excretory pore/tail length bigger than in the first generation. Tail conoid, with a slight postanal swelling (Fig. 2f).

#### Third juvenile stage

Body slender, habitus straight. Cuticle with fine transverse striae. Head continuous with body contour, slightly truncate (Fig. 3a), not annulated. Labial papillae not observed, amphidial opening like a pore at the level of four distinct cephalic papillae (Fig. 3b). Oral aperture and anus closed. Lateral fields with eight notorious ridges at midbody region (Fig. 3c). Long esophagous, narrow, procorpus slightly expanded, narrowing in isthmus and base bulb pyriform (Fig. 3a). Excretory pore at mid-esophagous level, isthmus surrounded by nerve ring. Deirids not observed. Cardia present. Small bacterium receptacle in the anterior part of intestine. Tail conoid, tapering gradually (Fig. 3d, e), hyaline portion equivalent to 36% of tail length (Fig. 3d).

### Molecular characterization

**Fig. 4:**
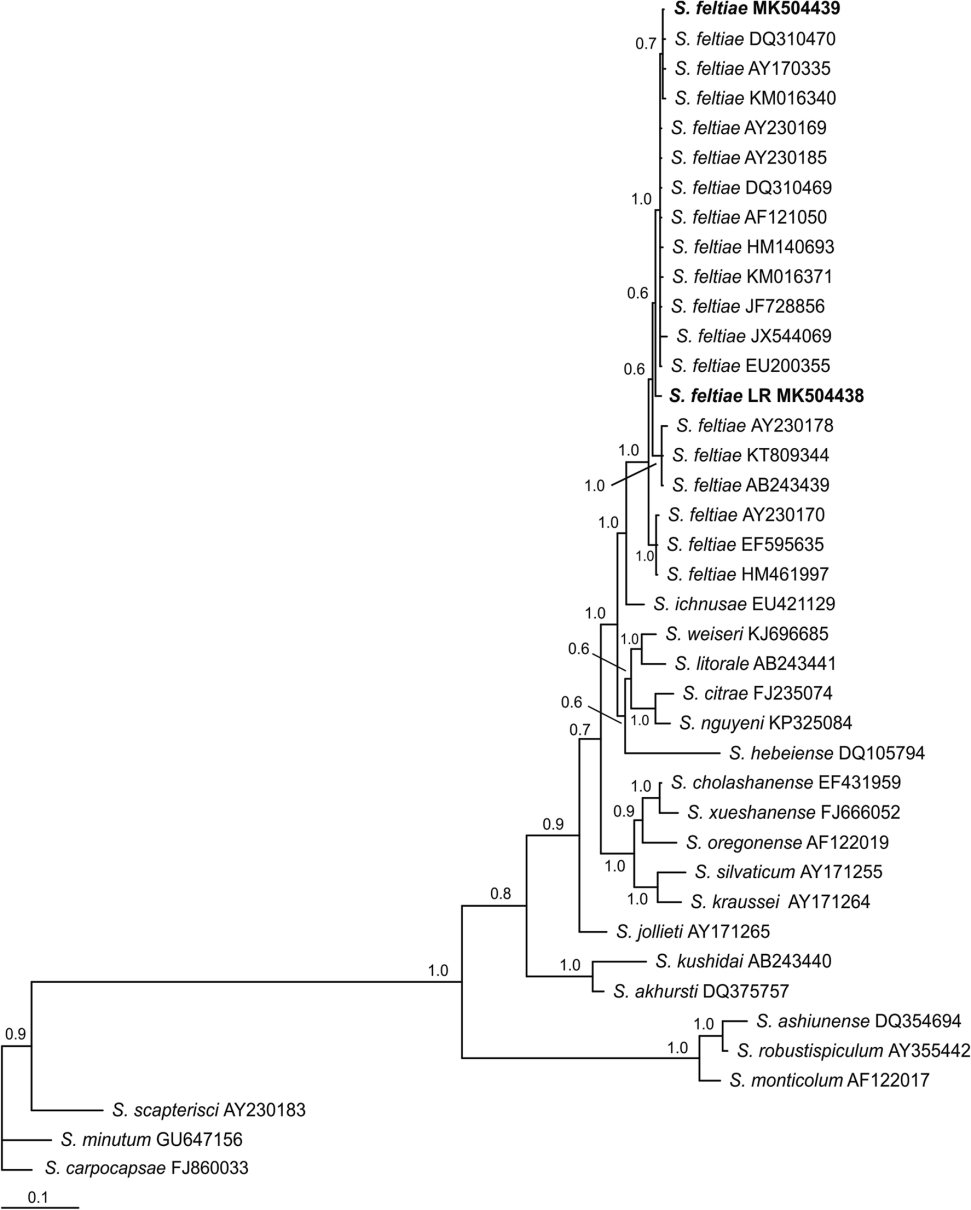
Phylogenetic relationships of the ITS rRNA sequences of *Steinernema* spp. The 50% majority rule consensus tree from Bayesian analysis generated with the GTR+G model. Posterior probabilities are given in the nodes. Newly obtained sequences are in bold letters

**Fig. 5:**
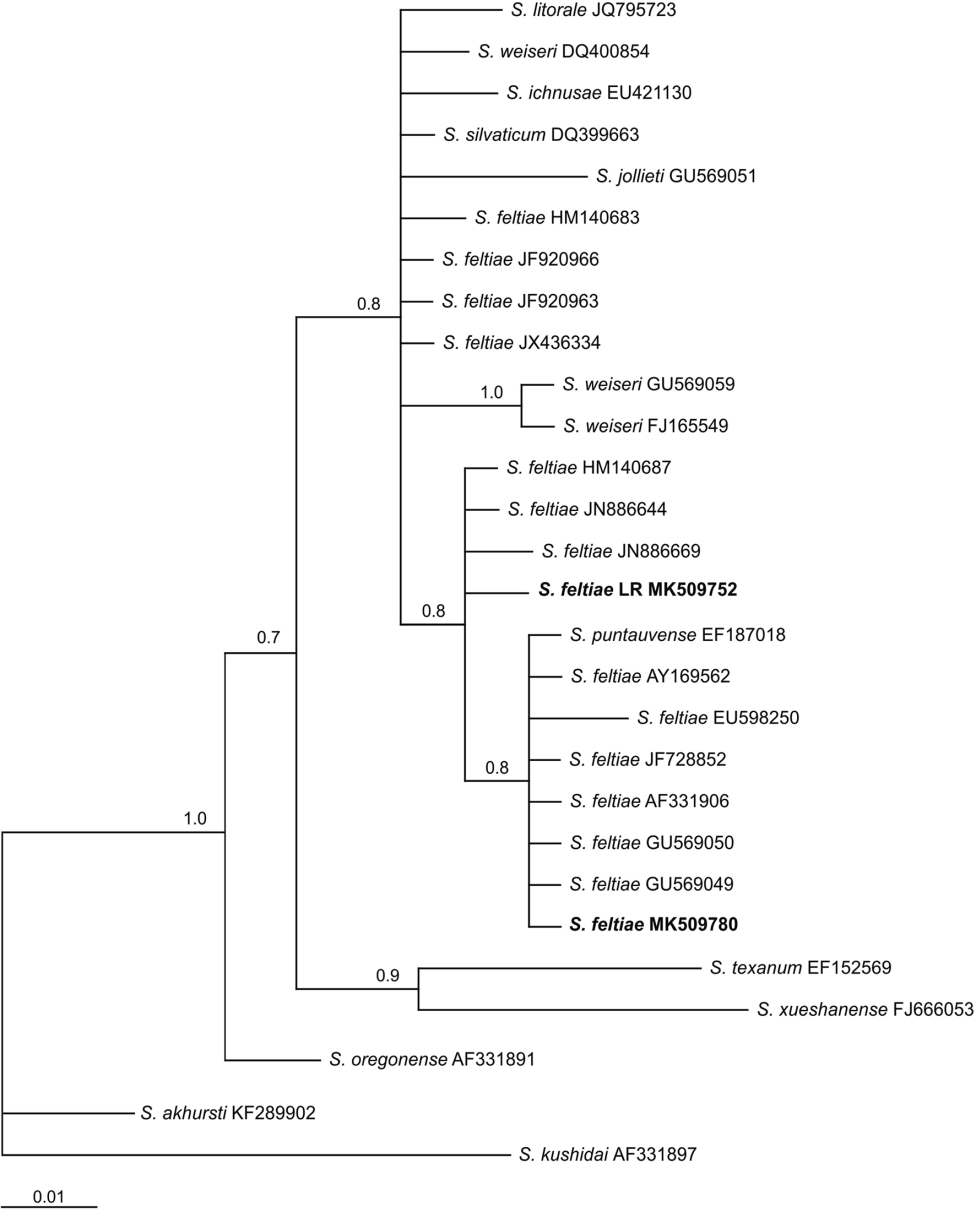
Phylogenetic relationships of the 28S rRNA sequences of *Steinernema* spp. The 50% majority rule consensus tree from Bayesian analysis generated with the GTR+G model. Posterior probabilities are given in the nodes. Newly obtained sequences are in bold letters

**Fig. 6:**
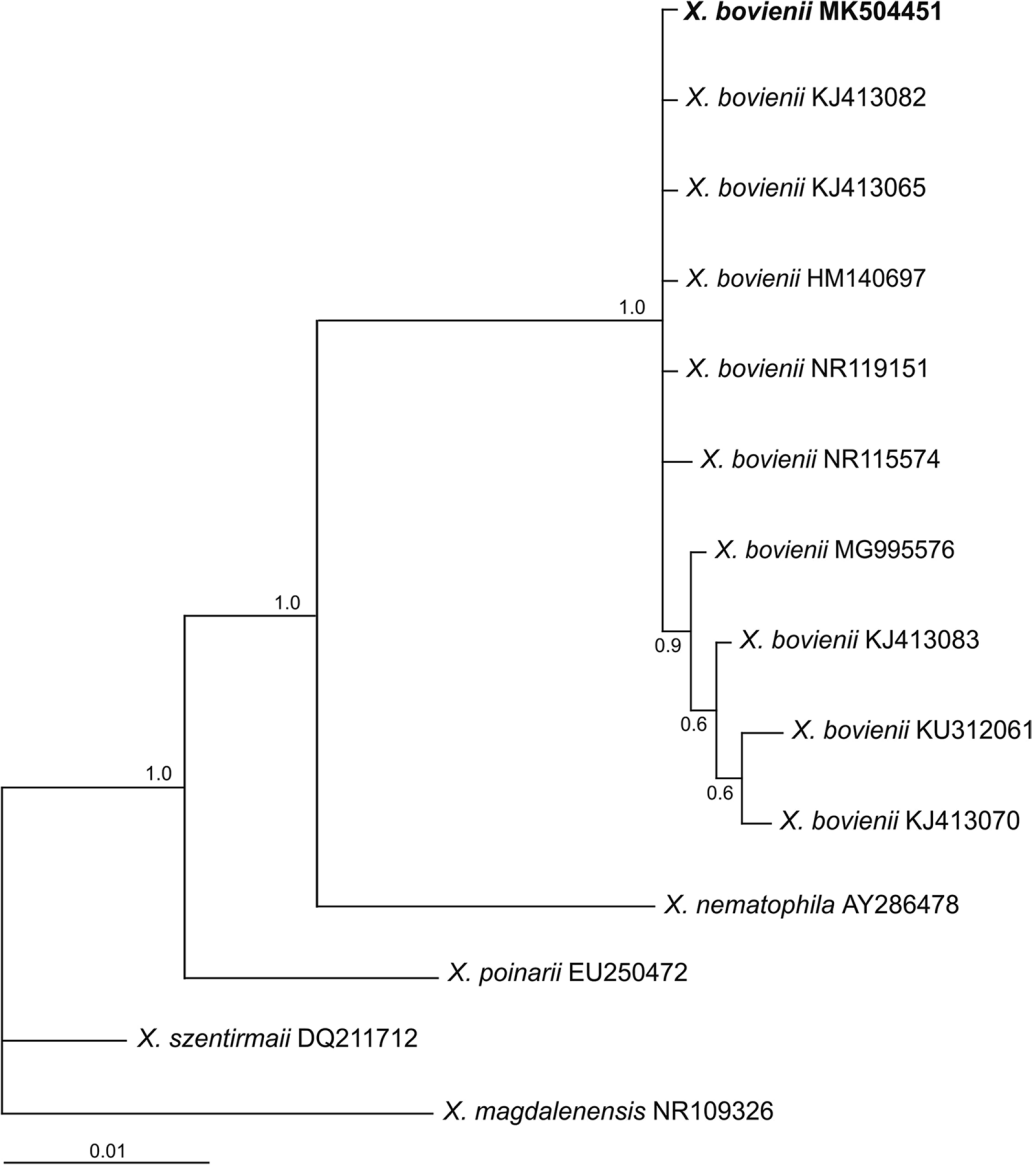
Phylogenetic relationships of the 16S rRNA sequences of *Xenorhabdus* spp. The 50% majority rule consensus tree from Bayesian analysis generated with the GTR+G+I model. Posterior probabilities are given in the nodes. Newly obtained sequences are in bold letters

For the ITS region a fragment of 859 bp was obtained for the Chilean *S. feltiae* isolate. This sequence was tested in BLAST with data deposited in GenBank, showing approximately 97–99% similarity with sequences of the same species. The majority-rule consensus tree of the Bayesian inference showed a well-supported group (100% bootstrap) that comprised the LR isolate (MK504438) and known sequences of *S. feltiae* from different countries, including one from Chillán, Chile (MK504439), sequenced in the present work as reference (Fig. 4). For 28S, a fragment of 894 bp was obtained (MK509752) showing 99% similarity with published sequences of *S. feltiae* and other species from the *feltiae* group. The phylogenetic relationships revealed a clade (100% bootstrap) that included sequences of *S. feltiae* from different geographical origins including Chillán, Chile (MK509780), and from *Steinernema jollieti, Steinernema puntauvense, Steinernema litorale, Steinernema ichnusae, Steinernema weiseri* and *Steinernema silvaticum* (Fig. 5). Based on the BLAST search and phylogenetic analysis of 16S rRNA, the symbiotic bacterium of *S. feltiae* LR is *Xenorhabdus bovienii* (BLAST similarities 99%). The Bayesian inference showed that the sequence obtained (MK504451) formed a well-supported group with sequences of the same species deposited in GenBank (Fig. 6). ML analysis produced trees with the same topology for all the genes considered.

### Ecological characteristics

#### Observations of the cycle of LR isolate

The life cycle of the LR isolate was similar to those described for other *Steinernema* species. The IJs were able to kill *G. mellonella* larvae between the 1st and 2nd day after inoculation at 20 °C. Males and females of the first generation were present on the 3rd or 4th day. On the 5th day, the first IJs were observed in the insect cadaver. A second adult generation occurred between the 7th and 8th days. IJs emerged massively from the insect body on the 10th day. The color of insect larvae turned brown when they died.

#### Effect of temperature

The effect of IJs on *G. mellonella* mortality at different temperatures is shown in Table 2. On the 2nd day, most larvae incubated at 20 °C or higher were immobile and starting to change color. While at 5 °C, no mortality was recorded, at 10 °C, a few dead larvae (10%) were observed on day 5 after inoculation (DAI). Mortality increased dramatically with higher temperatures, reaching 100% at 15, 20 and 25 °C on the 3rd DAI. The last two temperatures seemed to be optimal for reaching the highest mortality in the shortest period, with a 90–97.5% mortality on the 2nd DAI. The highest temperature (30 °C) had an effect on mortality, reaching the maximum value on the 5th DAI.

**Table 2:** Percent mortality of *Galleria mellonella* larvae at different temperatures during the 5 days after inoculation with infective juveniles of *Steinernema feltiae* LR

Temperature (°C)	Days after inoculation				
	1	2	3	4	5
5	0 a^x^	0 a	0 a	0 a	0 a
10	0 a	0 a	0 a	0 a	10 a
15	0 a	0 a	100 c	100 b	100 b
20	0 a	97.5 c	100 c	100 b	100 b
25	0 a	90 c	100 c	100 b	100 b
30	12.5 b	60 b	90 b	95 b	100 b

^X^: Means (*n* = 20) in columns followed by the same letter do not differ significantly according to Tukey’s multiple range test (*P* < 0.05)

The number of invader IJs per *G. mellonella* larva at different temperatures is presented in Table 3. At the lowest evaluated temperatures (5 and 10 °C), nematode presence was not detected by dissection. IJ penetration was higher at 15 and 20 °C (approximately 25% of IJs inoculated) and significantly decreased as temperature increased. Females with eggs were observed on the 2nd day after larval death at 15, 20 and 25 °C. The time for emergence of IJs (Table 3) from the insect cadaver was optimal for medium temperatures (15 and 20 °C), coming out of the insect around the 10th DAI. At the lowest (5 and 10 °C) and highest (30 °C) temperatures, neither IJ emergence nor offspring were observed. The greatest IJ production occurred at 20 °C. The maximum IJs recovery occurred during the 5th DAI, and the emergence lasted for 30 days (data not shown). The optimal temperature for host invasion and reproduction of *S. feltiae* LR was 20 °C, so this temperature was used for the following assays.

**Table 3:** Number of invader infective juveniles (IJs) per *Galleria mellonella* larva, time to emerge from the cadaver and offspring production (IJs/larva) after inoculation at different temperatures

Temperature (°C)	Invader IJs	Emergence days	Offspring
5	nd	nd	nd
10	nd	nd	nd
15	26.6 ± 11.4 c^x^	17.3 ± 1.4 c	72,884.9 ± 26,417 b
20	26.4 ± 11.2 c	10.6 ± 1.4 b	102,807.3 ± 23,256 c
25	12.4 ± 7.2 b	9.1 ± 0.7 a	52,107.8 ± 15,452 a
30	3.1 ± 2.6 a	nd	nd

nd: nematodes no detected

^X^: Means (*n* = 20) in columns followed by the same letter do not significantly differ according to Tukey’s multiple range test (*P* < 0.05)

#### Lethal concentration

Insect mortality percentages obtained during 4 DAIs are presented in Table 4, showing that mortality increased as the inoculum and DAI increased. On the 3rd day, 100% mortality was reached for all nematode doses; however, on the 2nd day, over 80 IJs were enough to achieve maximum control. The CL_50_ and CL_90_ at 48 h were 7.2 and 40.4 IJs/larva, respectively.

**Table 4:** Percentage of mortality of *Galleria mellonella* larvae during the 4 days after inoculation with different infective juvenile (IJs) doses at 20 °C

Doses (IJs/larva)	Days after inoculation			
	1	2	3	4
10	0 a^x^	70 a	100 a	100 a
20	0 a	70 a	100 a	100 a
40	0 a	80 ab	100 a	100 a
80	0 a	100 b	100 a	100 a
120	0 a	100 b	100 a	100 a
240	20 b	100 b	100 a	100 a

^X^: Means (*n* = 20) in columns followed by the same letter do not significantly differ according to Tukey’s multiple range test (*P* < 0.05)

The number of nematodes invading the host increased with dose (Table 5); however, efficacy of penetration, ranging from 24 to 49.6%, did not show statistical differences. Independent of IJ inoculum dose, no significant differences were observed respect to the time needed for new IJs to start to emerge from the insect cadaver, varying between 11 and 13.5 days. Differences were observed in the offspring emerging from the host, where doses of 120 IJs/larva were the most prolific. This nematode dose was used to investigate effects of soil water content.

**Table 5:** Number of invader infective juveniles (IJs) per *Galleria mellonella* larva, percentage of penetration efficacy, emergence time and offspring (number of IJs) at 20 °C and different doses of *Steinernema feltiae* LR

Doses (IJs/larva)	Invader IJs	Penetration efficacy	Emergence time (days)	Offspring
10	2.4 ± 1.7 a^x^	24 ± 16.7 a	13.5 ± 5.7 a	64,919.3 ± 41,294.8 a
20	6.6 ± 1.8 b	33 ± 9.1 a	12 ± 1.7 a	79,130.2 ± 16,801.7 ab
40	10 ± 5.2 bc	25 ± 13.3 a	11.2 ± 0.5 a	95,787.8 ± 18,570.1 abc
80	20.8 ± 3.9 d	26.4 ± 4.8 a	11.4 ± 0.6 a	90,316.2 ± 8542.4 abc
120	40.8 ± 17.3 de	34 ± 14.3 a	11 ± 0.7 a	106,152 ± 13,569.3 c
240	118.8 ± 46.8 f	49.6 ± 19.6 a	11.2 ± 1.5 a	86,240 ± 67,172 abc

^X^: Means (*n* = 5) in columns followed by the same letter do not significantly differ according to Tukey´s multiple range test (*P* < 0.05)

#### Effect of soil water content

The effect of soil water content on the mortality of *G. mellonella* over 5 days is shown in Table 6. On the 2nd and 3rd days, differences were observed between water contents; however, on the 4th day, mortality was statistically similar for all treatments, showing a delay in achieving full mortality. Water content did not affect the number of IJ invaders or penetration efficacy (Table 7). The time of emergence of the IJs and offspring per larva showed no differences between treatments.

**Table 6:** Assessment of mortality (%) of *Galleria mellonella* larvae during 5 days after inoculation with *Steinernema feltiae* LR infective juveniles (IJs) in soil with different water contents

Water content	Days after inoculation				
	1	2	3	4	5
Permanent wilting point	0 a^x^	73 a	93 a	98 a	100 a
Field capacity	2.5 a	93 b	100 b	100 a	100 a
Saturation	2.5 a	100 b	100 b	100 a	100 a

^X^: Means (*n* = 20) in columns followed by the same letter do not differ according to Tukey’s multiple range test (*P* < 0.05)

**Table 7:** Incidence of soil water content in number of invader infective juveniles (IJs) per insect larva, percentage of penetration, emergence days and offspring (number of IJs) of *Steinernema feltiae* LR

Water content	Invader IJs	Penetration efficacy	Time of emergence	Offspring
Permanent wilting point	24.9 ± 11.5 a^x^	20.8 ± 11.5 a	10.1 ± 1.6 a	75,180.1 ± 37,210.5 a
Field capacity	36.8 ± 21.4 a	30.8 ± 21.4 a	10.5 ± 0.7 a	81,518.3 ± 24,150.1 a
Saturation	38.4 ± 18.2 a	38.4 ± 18.2 a	10.1 ± 0.2 a	70,975.5 ± 23,629.5 a

^X^: Means (*n* = 20) in columns followed by the same letter do not differ according to Tukey´s multiple range test (*P* < 0.05)

## Discussion

Morphometric characteristics of *S. feltiae* showed a great intraspecific variability in mean values and ranges between isolates from different geographical origins [35–41]. In Chile, Edgington et al. [9] collected several isolates of this species from different zones (3, 4 and 6), and, based on ITS region, they were separated into two subgroups (I and II). Morphometric characters of IJs from isolate D030 (subgroup I) were smaller than from isolate D087 (subgroup II); the isolate LR, collected from zone 4, showed intermediate sizes for IJs.

Considering the high morphometric variability of *S. feltiae*, molecular data are very useful [36]; however, a marker such as the ITS region, which has proven useful in resolving phylogenetic relationships in EPNs, has exhibited intra-specific and intra-individual variability for this species as well as others in the *feltiae* and *glaseri* group [42]. According to these authors, considering the frequency of intra-individual variability, sequencing of the D2-D3 region of 28S appears to be necessary to confirm species status. However, this region is also conserved among some species of the *feltiae* group, as was observed when analyzing the phylogenetic relationships in the present work; other species, such as *Steinernema weiseri* and *Steinernema puntauvense,* were grouped within the *S. feltiae* clade with 99% genetic similarity. On the other hand, analysis of the 16S rRNA gene allowed for the identification of *X. bovienii*, the symbiotic bacterium of *S. feltiae* [6]. This bacterium species, like other members of the genus, produces a toxin complex with a potential use in crop protection against different insect pests [4, 43].

*Steinernema feltiae* is a common species reported in many places in the world; this species is known to be adapted to cold, capable of infecting hosts in temperatures between 8 and 28 °C and able to produce offspring between 8 and 25 °C [44–47]. Based on climatic conditions from the site of origin (mean temperature of 8 °C and 17.7 °C in winter and summer, respectively), *S. feltiae* LR could be an isolate more adapted to cold conditions. However, below 10 °C, the IJs were not able to infect *G. mellonella*, and 15–25 °C was the optimum temperature range for infection and reproduction. This finding could indicate that the isolate’s parasitic activities under natural conditions are higher in spring–summer. The optimal temperature ranges vary with the species and/or the isolate. Studies performed by Umana [48] with seven isolates of *S. feltiae* obtained from Chile [9] showed that at 20 °C, *G. mellonella* death occurred at 48–72 h, and the output of new larvae started within 13–14 days. At the same temperature, the isolate LR achieved highest larval mortality, and the number of days for IJ emergence was 3–4 days shorter.

Sáenz [49] considered that the mortality of *S. feltiae* is not dependent on the number of IJs penetrating the host, but Fan et al. [50] estimated that mortality increases with the amount of inoculum. According to our results, the number of nematodes able to enter the host increased with the number of IJs inoculated, but the efficacy of penetration was similar; this observation was also reported by Fan et al. [50] with *S. feltiae*, who obtained 100% mortality with an initial population ranging from 53 to 114 IJs per *G. mellonella* larva, and the penetration capacity was variable; about 20–50% of applied IJs penetrated the host. According to Lewis et al. [7], there is a minimum number required to overcome the insect’s defenses and a maximum due to a high competence between them. In our study, with 240 IJs per larva, the reproductive rate was minimum. It is also interesting that according to CL_90_, with 40.4 IJs/larva, there was no increase in mortality from the 2nd DAI, showing that over an inoculum dose threshold, mortality does not increase. Koppenhöfer and Kaya [51] observed that an increase in dose of *S. glaseri* in soil affected the penetration and reproduction in *G. mellonella* larvae. They observed that the highest number of new IJs occurred between 20.7 and 58 IJs per larva and that no reproduction was observed with 184.4 specimens or more.

Moisture is another important soil factor for survival and infectivity of NEPs, since they need a water film around them for movement [52–54]. The water content in soil may affect movement, penetration and other factors depending on the nematode species and their physiological adaptations. The optimal ranges are variable across different species [53, 55]. Infectivity and reproduction of *S. feltiae* LR were optimal when water content was near field capacity, similar to observations by Koppenhöfer and Fuzy [53] working with *S. scarabei* in sandy loam and loamy soils. Susurluk et al.  [56] reported that the ideal water content for *S. feltiae* host penetration was 10%, with drastic decreases when water content increased to 20%. Gungor et al. [55] found similar results with *Steinernema anatoliense*, reporting an optimum at 10% of water content. In our study, the amount of water in the soil affected only the time needed to reach 100% mortality, being necessary one more day in the driest substrate, but efficacy and offspring were the same for the three water contents evaluated.

According to the results obtained, the species *S. feltiae* LR has ecological requirements that would allow it to be used in various areas of the country, where the conditions required for optimal development occur.

## Conclusions

Due to their potential as biological control agents, the correct identification of EPNs and their symbiont bacteria, as well as the study of their ecological characteristics, is key for optimal management in pest control. Data have shown that *S. feltiae* LR shows certain variation in morphometric characteristic compared with other isolates from different geographic origins; molecular analysis also evidenced intra-specific variability. The LR isolate was shown to be efficient in water-containing soil, with optimal temperatures ranging from 15 to 25 °C for host infestation and production of an abundant offspring; these characteristics would allow its potential use as a control agent in a wide geographical area of the country.

## Data Availability

Our sequences were deposited in the GenBank database under the accession numbers MK504438 (ITS rRNA), MK504439 (ITS rRNA), MK509752 (28S rRNA), MK509780 (28S rRNA) and MK504451 (16S rRNA). Data supporting the conclusions of this article are included within the article. The datasets used and/or analyzed during the current study are available from the corresponding author upon reasonable request.

## Abbreviations

IJs: Infective juveniles
ITS: Internal transcribed spacer
EPNs: Entomopathogenic nematodes
LR: Lican Ray
NCBI: National Center for Biotechnology Information
LSU: Large ribosomal subunit
ML: Maximum likelihood
DAI: Days after inoculation
LC_50_: Lethal concentration 50
LC_90_: Lethal concentration 90
